# Smad gene expression in pulmonary fibroblasts: indications for defective ECM repair in COPD

**DOI:** 10.1186/1465-9921-9-83

**Published:** 2008-12-16

**Authors:** Andre Zandvoort, Dirkje S Postma, Marnix R Jonker, Jacobien A Noordhoek, Johannes TWM Vos, Wim Timens

**Affiliations:** 1 Department of Pathology, University Medical Center Groningen, University of Groningen, Hanzeplein 1, NL-9713 GZ Groningen, The Netherlands; 2 Department of Pulmonology, University Medical Center Groningen, University of Groningen, Hanzeplein 1, NL-9713 GZ Groningen, The Netherlands

## Abstract

**Background:**

Chronic Obstructive Pulmonary Disease (COPD) is characterized by defective extracellular matrix (ECM) turnover as a result of prolonged cigarette smoking. Fibroblasts have a central role in ECM turnover. The TGFβ induced Smad pathway provides intracellular signals to regulate ECM production. We address the following hypothesis: fibroblasts have abnormal expression of genes in the Smad pathway in COPD, resulting in abnormal proteoglycan modulation, the ground substance of ECM.

**Methods:**

We compared gene expression of the Smad pathway at different time points after stimulation with TGFβ, TNF or cigarette smoke extract (CSE) in pulmonary fibroblasts of GOLD stage II and IV COPD patients, and controls.

**Results:**

Without stimulation, all genes were similarly expressed in control and COPD fibroblasts. TGFβ stimulation: downregulation of Smad3 and upregulation of Smad7 occurred in COPD and control fibroblasts, indicating a negative feedback loop upon TGFβ stimulation. CSE hardly influenced gene expression of the TGFβ-Smad pathway in control fibroblasts, whereas it reduced Smad3 and enhanced Smad7 gene expression in COPD fibroblasts. Furthermore, decorin gene expression decreased by all stimulations in COPD but not in control fibroblasts.

**Conclusion:**

Fibroblasts of COPD patients and controls differ in their regulation of the Smad pathway, the contrast being most pronounced under CSE exposure. This aberrant responsiveness of COPD fibroblasts to CSE might result in an impaired tissue repair capability and is likely important with regard to the question why only a subset of smokers demonstrates an excess ECM destruction under influence of cigarette smoking.

## Introduction

Chronic Obstructive Pulmonary Disease (COPD) is a severe, slowly progressive and disabling disease associated with accelerated lung function decline. COPD consists of emphysema, small airways disease, and chronic bronchitis, which may be present alone or in combination of different intensities. Emphysema is due to an extensive extracellular matrix (ECM) destruction of lung parenchyma [1-5]. In addition, emphysema can coincide with fibrosis of the airways as observed in chronic bronchitis and small airways disease [1,6-8]. The generally held hypothesis is that cigarette smoke induces an excess in extracellular matrix (ECM) degrading enzymes and reactive oxygen species that subsequently lead to ECM destruction of lung parenchyma [1]. We here propose a third important contributing factor to COPD development, i.e. an aberrant fibroblast function that contributes to disturbance of ECM homeostasis.

**Figure 1:**
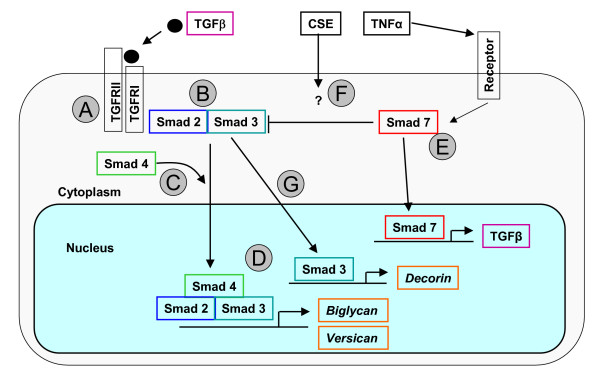
Simplified schematic drawing of the TGF-β-Smad pathway. Binding of TGF-β to its type II receptor in concert with the type I receptor (A) leads to formation of a receptor complex and phosphorylation of the type I receptor. The type I receptor subsequently phosphorylates Smad2 or 3 (B), allowing this complex to associate with Smad4 and move into the nucleus (C). In the nucleus, the Smad complex associates with a transcription factor and this complex binds to specific enhancers in target genes (down-) regulating transcription (D). TNF is able to interfere with TGF-β signaling through the upregulation of the inhibitory Smad7 protein (E). Smad7 is capable of inhibiting the Smad2 and 3 phosphorylating process by competing with the receptor interaction but Smad7 also can dephosphorylate the complex. In addition, Smad7 itself is capable to upregulate TGFβ gene expression. As described in the discussion, CSE is most likely capable to interfere with the Smad pathway although this is not yet fully elucidated. (F). Phosphorylated Smad3 is able to stimulate the transcription of the decorin gene (G). Adapted from [18,19,35,36].

Pulmonary fibroblasts are essential cells in tissue repair processes since they are key producers of ECM constituents [9]. TGFβ is the main cytokine that stimulates fibroblasts to produce ECM constituents like decorin, biglycan, versican, and collagens [10]. The production of ECM components is inhibited by pro-inflammatory cytokines like TNF and IFN-gamma [11]. We have demonstrated previously that lung tissue of COPD patients contains less decorin, an important proteoglycan of the ECM [9,12]. In addition, our studies have shown that pulmonary fibroblasts of COPD patients with GOLD stage IV (very severe COPD[13]) produce less decorin after TGFβ stimulation than those of controls [14]. We hypothesize that this results from a defect or alteration in gene expression of the Smad pathway, since this regulates transcription of ECM proteins like collagens, decorin, versican and biglycan [11,15-17]. Smads are intracellular signal transducers transporting the TGFβ activation signal from the receptor to the nucleus in order to initiate gene transcription of ECM constituents [11,18-20] (Figure 1). TGFβ interacts with the TGFβ receptor II which in turn activates TGFβ receptor I. This activated TGFβ receptor complex induces phosphorylation of the Smad2-Smad3 complex that can interact with the transporter Smad4. The resulting complex enters the nucleus and initiates gene transcription of ECM constituents. Smad7 on the other hand can affect this pathway by inhibiting phosphorylation of Smad2 and Smad3 and inducing ubiquination of their receptors. Additionally, Smad7 can induce TGFβ-production by fibroblasts upon activation by pro-inflammatory cytokines like TNF and IFN-gamma, thereby enabling autocrine stimulation that counterbalances superfluous Smad7 effects [21].

Ideally, fibroblasts should be capable of repairing cigarette smoke induced lung damage. This repair process appears inadequate in a subset of smokers, leading to gradually increasing parenchymal ECM destruction and emphysematous changes [9]. We addressed three questions to obtain more insight in the underlying mechanisms of this selective susceptibility, following our hypothesis that the Smad pathway is involved. Because of the limited knowledge on gene modulation of the Smad pathway we first addressed the question how Smad pathway genes are regulated in control fibroblasts under influence of TGFβ, TNF and cigarette smoke components. We compared fibroblasts of COPD patients and controls as to their basal expression of Smad pathway genes. Finally, we investigated whether TGFβ, TNF and CSE, stimuli that are of prime importance to fibroblast functioning [1,2,22,23], stimulate the Smad pathway differently in primary fibroblast cultures of COPD patients and controls. To analyze Smad gene expression, fibroblasts were harvested 1 hour, and 24 hours after incubation with TGFβ, TNF or CSE. Expression of Smad pathway related genes were analyzed by real-time PCR. As functional read-out of Smad gene regulation, we determined gene expression of decorin, versican, and biglycan (Figure 1), ECM proteoglycans that are relevant to the development of COPD.

## Patients and methods

### Study design

To answer the question whether genes of the Smad pathway are differentially expressed in COPD and controls, we stimulated primary fibroblasts of patients with stage II and stage IV COPD and controls with TGFβ, TNF and cigarette smoke extract (CSE) for 1, and 24 hours. Primary fibroblasts were cultured from lung tissue, obtained from 23 individuals. Classification of COPD severity was based on the 2003 Global initiative for chronic obstructive lung disease (GOLD) criteria [13]. Three groups were made: Individuals with moderate (GOLD stage II, n = 9), and with very severe COPD (stage IV, n = 9), and individuals with histological normal lungs (n = 5). Patients did not show clinical signs of chronic bronchitis and were not α_1_-antitrypsin deficient. Emphysema was assessed by routine histological examination of lung tissue, performed by an experienced pulmonary pathologist (WT). The clinical characteristics of the groups are presented in table 1. Informed consent was obtained from all patients. The study protocol was consistent with national ethical and professional guidelines ("Code of Conduct; Dutch Federation of Biomedical Scientific Societies"; ).

**Table 1:** Characteristics of the control and patient groups

	*Control*	*Stage II*	*Stage IV*
Number of subjects	5	9	9
Age (years)	62 (36–63)	70 (44–81)	55 (44–61)
Smoking (Ex/C)	2/3	5/4	9/0
Pack-years	13 (0–75)	30 (8–58)	30 (17–54)
FEV_1 _% predicted	86 (83–108)	73 (50–76)	19 (13–29)
FEV_1_/FVC % pred	73 (70–81)	55 (37–68)	35 (24–66)

All values are presented as median values with ranges in parentheses. Ex = ex smokers, not smoking for at least one year, C = current smokers; FEV_1 _% predicted = Forced Expiratory Volume in 1 second as percentage of predicted value; FVC = forced vital capacity; pred = predicted. Stage means severity of COPD according to GOLD criteria.

Tissue of GOLD stage II COPD patients (median FEV_1 _73% of predicted) was derived from non-involved lung tissue from patients undergoing resective surgery for pulmonary carcinoma. Tissue was always taken as far as possible from the tumor, or from a non-involved lobe. Histopathologically emphysematous lesions were present, yet of limited but varying severity. The moderate forms can be histopathologically demonstrated by the finding of isolated or free-lying segments of viable alveolar septal tissue or isolated cross sections of pulmonary vessels [19,20].

Tissue of GOLD stage IV COPD patients (median FEV_1 _19% of predicted) was obtained from COPD patients undergoing surgery for lung transplantation or lung volume reduction. All individuals had quitted smoking for at least 1 year before surgery. The resected tissue showed both macroscopically and microscopically severe emphysematous lesions, often accompanied by bullae. Sub-pleural fibrous areas were avoided.

Tissue of the control group (median FEV_1 _86% of predicted) was derived from non-involved lung tissue from patients undergoing resective surgery for pulmonary carcinoma. Patients had no airway obstruction, nor chronic airway symptoms like cough and sputum production. Material was always taken as far as possible from the tumor, or from a non-involved lobe. No histopathological abnormalities were present.

### Isolation and culture of lung fibroblasts

Pulmonary fibroblast cultures were established from parenchymal lung tissue by explant technique. Absence of mycoplasma contamination in the fibroblast cultures was confirmed with a mycoplasma detection kit (Roche Diagnostics, Almere, The Netherlands). Isolated cells were characterized as fibroblasts by morphological appearance and expression pattern of specific proteins as described previously [24]. Fibroblast cultures were stored into liquid nitrogen until use.

Fibroblast cultures cultured in complete culture medium (Ham's F12, 10%FBS, penicillin, streptavidin and glutamin (all from Cambrex, Verviers, Belgium)). Experiments were performed on fibroblasts of passage 5/6 with confluent growth. After reaching confluence, fibroblasts were cultured for 24 h on 0.5% FBS culture medium before the stimulations started. Cells were washed with Ham's culture medium without FBS and incubated with the appropriate stimulus, diluted in complete culture medium. TGFβ (R&D systems, Abingdon, UK) was used in a concentration of 100 u/ml, TNF (R&D systems) in a concentration of 1000 u/ml and CSE was used in a concentration of 2.5%. CSE was prepared according to a standardized protocol by bubbling the puffs of 4 cigarettes (Kentucky University research cigarettes) through 50 ml of Ham's F12 (Cambrex). The medium was filter sterilized with a 22 um filter. After the designated duration of the stimulation (1 and 24 hours) cells were harvested by trypsinization (Trypsin, Cambrex) followed by two wash steps. Non-stimulated fibroblasts were also harvested at 1 h and 24 h for comparison of effect of stimulation. Cell pellets were lysed using lysis buffer of the RNeasy mini kit for RNA isolation (Qiagen, Hilden, Germany). Optimal concentrations and durations of the stimulation were determined in previous experiments (data not shown). As a check for an adequate effect of TGFβ stimulation, expression of plasminogen activator inhibitor (PAI)-1, being a classical TGFβ regulated gene, was analyzed, similar to the other genes. PAI-1 was significantly upregulated by TGFβ in all fibroblast samples tested.

### Real time PCR

Fibroblast total RNA was isolated using the RNeasy mini kit (Qiagen). RNA quantity and quality (OD 260/280) were determined by optical density measurements on the Nanodrop. Total RNA was treated with DNase I during RNA isolation and run over the column to remove genomic DNA (Qiagen RNA-se free DNAse set). Three ng mRNA was transcribed into cDNA by reverse transcriptase II (Invitrogen, Breda, the Netherlands). Real time PCR was performed on an ABI7900 HT sequencer with "Assay on Demands" from Applied Biosystems (Foster City, CA, USA), according to the manufacturers' instructions. Expression of the following genes was analyzed: Smad2, 3, 4, 7, versican, biglycan, and decorin. Data were analyzed by the delta-delta-Ct method [25]. In brief, concentration in time (Ct) values of the genes of interest were corrected for Ct values from a housekeeping gene, resulting in a delta-Ct value. In case of stimulation, the obtained delta-Ct was normalized to the delta-Ct of the non-stimulated sample value at the same time point (delta-delta-Ct). The 2^-delta-delta-Ct ^was taken for each stimulated fibroblast sample, which was compared to its unstimulated counterpart and presented as a percentage of this basal value; each basal values was set to 100%. Several housekeeping genes were tested for the influence of the experimental procedure on the expression. Ribosomal protein S9 was chosen as most optimal household gene because gene expression was most stable under basal as well as stimulation conditions.

### Statistical analysis

Differences in subject characteristics and real time data between the gene expression at basal levels were analyzed using the Kruskal-Wallis test followed by the Mann-Whitney U test. Differences between gene expression after stimulation were analyzed using the Wilcoxon signed rank test. To analyze differences between COPD- and control fibroblasts, we calculated the percent change from the stimulated value as compared to the basal value. Significant differences in this percent change between COPD fibroblasts and controls were also analyzed using the Kruskal-Wallis test followed by a Mann-Whitney U test. The level of significance used was < 0.05, all reported P-values are two-sided.

## Results

### Influence of TGFβ, TNF, and CSE on Smad pathway related gene expression in fibroblasts

#### Healthy individuals

##### TGFβ stimulation

**Figure 2:**
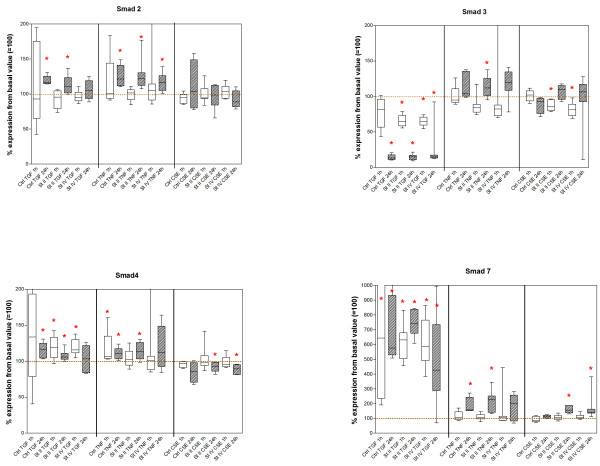
Smad2, 3, 4 and 7: Results of real-time PCR analysis at 1 and 24 h, presented for control and disease stage, per stimulation. Values are based on 2^-delta-delta-Ct ^values and represent the median percentages compared to the basal value before each stimulation (basal set to 100%). Asterisks indicate significant change compared to basal values. St II = GOLD stage II, St IV = GOLD stage IV.

**Figure 3:**
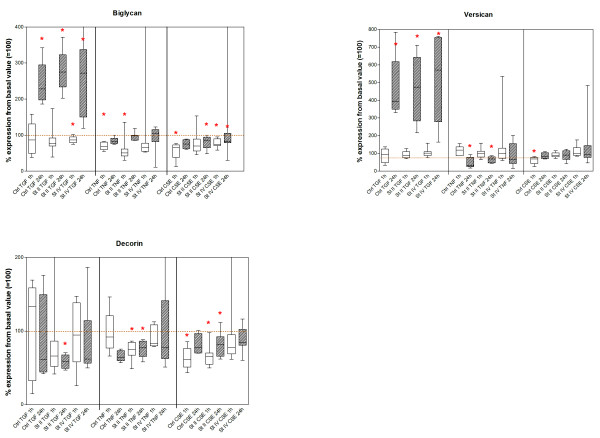
Decorin, biglycan and versican: Results of real-time PCR analysis at 1 and 24 h, presented for control and disease stage, per stimulation. Values are based on 2^-delta-delta-Ct ^values and represent the median percentages compared to the basal value before each stimulation (basal set to 100%). Asterisks indicate significant change compared to basal values. St II = GOLD stage II, St IV = GOLD stage IV.

Downregulation of Smad3 gene expression occurred at 24-hour stimulation, while Smad2, Smad4, biglycan and versican gene expression were upregulated (figures 2 and 3; additional file 1). Smad7 gene expression was upregulated by TGFβ at both 1-hour and 24-hour stimulation.

##### TNF stimulation

Upregulation of Smad2 and 7 gene expression occurred at 24-hour stimulation. Smad4 was upregulated at both 1-hour and 24-hour stimulation. Biglycan was downregulated after 1 hour. Versican gene expression was downregulated at 24-hour stimulation (figures 2 and 3).

##### CSE

No effect on Smad genes occurred. The genes for biglycan, decorin, and versican were downregulated after 1 hour of exposure, without an effect at 24 hours.

#### COPD GOLD stage II patients

##### TGFβ stimulation

Whereas Smad3 gene expression in COPD stage II fibroblasts was significantly downregulated at 1- and 24-hour stimulation (figures 2 and 3; additional file 1), Smad4 and Smad7 gene expression was upregulated, Smad2 was upregulated only at 24 hours. Decorin gene expression was significantly downregulated at 24-hour stimulation, whereas biglycan and versican gene expression was upregulated at 24 hours.

##### TNF stimulation

All Smads were significantly upregulated at 24 hours. Decorin expression was downregulated at both 1- and 24-hour stimulation. Biglycan gene expression was downregulated at 1 hour and versican gene expression was downregulated at 24 hours.

##### CSE exposure

Smad3 and decorin gene expression was downregulated at 1 hour. Smad4, decorin and biglycan gene expression was downregulated at 24 hours. Smad7 gene expression was upregulated at 24-hour stimulation.

### COPD GOLD stage IV patients

#### TGFβ stimulation

Smad3 gene expression was downregulated at both time points, while Smad7 gene expression was upregulated; Smad4 was upregulated at 1 hour. Biglycan gene expression was downregulated at 1 hour, whereas biglycan and versican gene expression was upregulated at 24-hour TGFβ stimulation.

##### TNF stimulation

TNF resulted in upregulation of Smad2 gene expression at 24-hour stimulation.

##### CSE exposure

CSE exposure resulted in a downregulation of Smad3 gene expression after 1 hour of stimulation, while biglycan gene expression was also downregulated at that time point. Smad4 and biglycan were downregulated and Smad7 gene expression was upregulated at 24 hours after CSE exposure.

### Differences in Smad pathway gene expression between COPD and control fibroblasts

**Figure 4:**
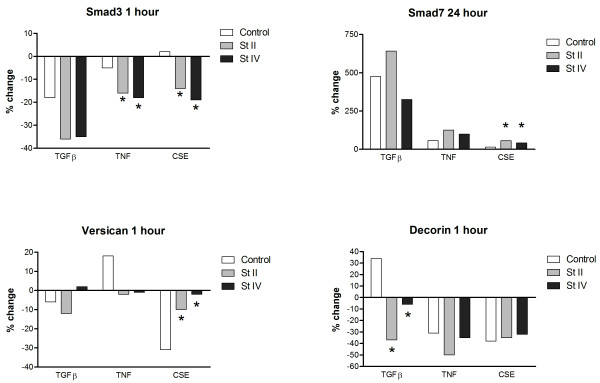
Differential gene expression in COPD fibroblasts compared to control fibroblasts. X-axis shows different stimuli, y-axis shows the percentage change of the 2^-delta-delta-Ct ^values from the stimulation compared to basal expression. Basal gene expression did not significantly differ between stage II, stage IV, or control fibroblasts for each of the different genes and time points. * indicates a significant difference between COPD and control fibroblasts.

To specifically analyze the differential expression between COPD fibroblasts and control fibroblasts, we calculated the percentage *change *in gene expression after stimulation from its basal value for each subject group. Subsequently we checked for differences in strength of the stimulation effect between controls and COPD fibroblasts by determining the difference between the change in gene expression from control fibroblasts when compared to stage II and IV COPD fibroblasts, respectively (figure 4).

Without stimulation, gene expression levels were similar between COPD and control fibroblasts. Smad2 and Smad4 gene expression showed no differential expression between COPD and control fibroblasts at all. Genes that were differentially expressed between COPD and control fibroblasts after stimulation with TGFβ, TNF or CSE are depicted in figure 4. Smad3 gene expression decreased significantly more in fibroblasts from COPD patients than from controls at 1 hour after TNF as well as CSE exposure (figure 4A). Smad7 gene expression was significantly more increased in COPD than control fibroblasts after 24 hours CSE exposure (figure 4B). Versican gene expression was significantly less decreased in COPD Stage II and IV than control fibroblasts at 1 hour after CSE exposure (figure 4C). Decorin gene expression was significantly more reduced in stage IV than the other group fibroblasts after TGFβ-stimulation, whereas an increase was observed in controls (figure 4D).

## Discussion

We addressed the question whether genes of the Smad pathway are aberrantly expressed in COPD fibroblasts with and without TGFβ, TNF, and cigarette smoke extract (CSE) stimulation. We provide suggestive evidence that this is indeed the case. Since the Smad pathway coordinates a delicate balance between fibrosis and excess ECM destruction, alterations in this pathway can contribute to the development of COPD. All investigated genes were similarly expressed in control and COPD fibroblasts without stimulation. In contrast, we observed a stimulus dependent gene expression of the Smad pathway with differential expression in COPD and control fibroblasts. In particular Smad3 and Smad7 were aberrantly regulated in COPD fibroblasts under influence of TNF and CSE (figure 4). Moreover, the ECM proteoglycans decorin and biglycan were downregulated in COPD fibroblasts after 24 hours of CSE exposure.

Little is known about the regulation of Smad and ECM gene expression in lung tissue fibroblasts of COPD patients by TGF-β, TNF, and CSE, factors that are of prime importance in COPD development. We therefore first investigated the regulation of the Smad pathway in control fibroblasts and observed that TGFβ stimulation in general resulted in downregulation of Smad3 gene expression while Smad2, 4, 7, biglycan and versican gene expressions were upregulated. This downregulation of Smad3 gene expression by TGFβ, together with the upregulation of Smad7 supports an inhibitory feedback mechanism because a reduced presence of Smad3 mRNA will result in reduced levels of Smad3 protein, as suggested previously [16]. Surprisingly, downregulation of Smad3 gene expression did not result in direct downregulation of ECM gene expression, but in contrast biglycan and versican gene expression was upregulated. This may be due to the fact that there is sufficient Smad3 protein available within the measured period of stimulation which subsequently activates ECM gene expression.

An unexpected finding was that TNF induced upregulation of Smad2, 4 and 7 mRNA in control fibroblasts after 24-hrs, similar to TGFβ. Furthermore, TNF induced an early downregulation of biglycan and later downregulation of versican. TNF has been demonstrated to be able to inhibit ECM production by fibroblasts [26]. This is in accordance with the reduced biglycan and versican gene expression in our control fibroblasts. Based on our previous findings we can conclude that TGFβ is known [11] to have a stimulatory effect on the Smad pathway at the protein level, yet this effect is less clear for Smad-gene expression. As Smad proteins are rather stable intracellular proteins, stimulation of the Smad pathway may result in downstream increased production of extracellular matrix proteins without necessarily a direct upregulation of Smad-gene expression. However, the increased ECM protein end-product may lead to a negative feedback loop that affects the gene expression level. Furthermore, also the effect on the balance between the stimulating and inhibiting Smad gene products is of importance in relation to the actual resulting ECM production.

The second research question we addressed is whether the Smad pathway is differentially expressed under basal conditions in fibroblasts of COPD patients and control subjects. We observed no significant differences between the groups suggesting that the altered modulation of the ECM as observed in COPD is not due to intrinsic differences in basal expression levels of the Smad pathway genes in fibroblasts. We can not rule out that the basal gene expression is normalized due to culturing of the fibroblasts for several passages. However, our data show that these fibroblasts are still capable of responding to stimuli and this particularly uncovers the differential fibroblast response in COPD patients and controls.

The third question we addressed is whether the differences in ECM modulation as observed in COPD can be ascribed to a differential modulation of the Smad pathway under influence of COPD relevant cytokines and cigarette smoke, the most important etiologic risk factor of COPD. Stage II and IV COPD fibroblasts displayed up- and downregulation of Smad genes under influence of TGFβ and TNF in the same direction as the control fibroblasts. As CSE exposure mainly induced inhibition of expression, it seems likely that a general effect may play a role, although of course some of the presumed > 5000 cigarette smoke compounds also may have a specific role affecting certain receptors or downstream mediators. Importantly, CSE exposure induced differential effects on Smad4 and 7, and biglycan and decorin gene expression of COPD fibroblasts, findings that were not or only transiently observed in control fibroblasts. Apparently, fibroblasts of COPD patients are more reactive to components of cigarette smoke extract. Of importance to the pathogenesis of COPD, CSE blocked the repair effect of COPD pulmonary fibroblasts, as represented by a decreased Smad3 gene expression at 1 hour and Smad 4 expression at 24 hour, together with the elevated Smad7 gene expression at 24 hours. It has been demonstrated that CSE is capable to upregulate the expression of GADD34, a cell cycle related protein [27]. The inhibitory effect of Smad7 on Smad2 and/or Smad3 phosphorylation is most likely mediated via GADD34 [28]. This indicates that CSE is capable to induce decreased Smad2 and Smad3 activation by dephosphorylation of the TGFβ receptor I and thus is indirectly capable of inhibiting the ECM gene transcription. This is compatible with the observation that Smad3 deficiency in mouse knock-out models results in abnormal lung alveolarization resembling emphysema [29,30]. We also found increased expression of Smad7, the inhibitor of ECM production that can also be involved in pro-inflammatory actions [31,32]. Thus, it seems likely that ongoing smoking in susceptible individuals leads to continuous suppression of Smad3 activation and continuous stimulation of Smad7 gene expression, explaining chronic local suppression of tissue repair.

In contrast to the increased Smad7gene expression in our lung tissue fibroblasts, we previously demonstrated a reduced expression of Smad7 protein in bronchial epithelial cells of COPD patients compared to control subjects [33]. In addition, Springer et al. demonstrated a reduced Smad7 gene expression in bronchial biopsies of stage II COPD patients. This may be indicative for the fibrotic processes observed in COPD airways [34]. Springer and coworkers incubated a bronchial epithelial cell line with CSE for 48 hours and found a reduced Smad7 under these conditions, and concluded that cigarette smoke is a reducing factor for Smad7 expression [34]. Apparently, Smad7 regulation is dependent on the cell type and/or on the duration of stimulation because we found an increase in Smad7 gene expression in our primary parenchymal fibroblasts after 24 hours under influence of CSE. Together, the results of their study and ours match the outcome of fibrosis in the airways and excess ECM destruction in the parenchyma as observed in emphysema.

Lung fibroblasts from COPD patients as well as controls showed an upregulation of biglycan and versican gene expression after 24-hour TGFβ stimulation. This indicates that lung fibroblasts of COPD patients are capable of upregulating ECM genes on gene expression level, and the results depend on the effects of the local cytokine microenvironment and the presence of cigarette smoke. Of interest, and compatible with our previous observations, decorin gene expression was mainly downregulated in COPD fibroblasts, a finding that occurred with all stimuli, and only transiently at 1 hour after CSE in control fibroblasts. This highlights the putative important role of decorin in COPD.

Obviously our data are a first set of experiments that now need expansion at the protein level and including additional kinetic data. There are some limitations to our study since our experiments were focused on intracellular regulation, and hence it is not always clear whether the direction of the modulation is a direct result of the applied stimulus or the result of a cellular counteraction, as a response to upregulation of other cellular factors by this applied stimulus. Therefore, our data is conclusive as to our hypothesis that there is an aberrant regulation of the Smad pathway, but it is only supportive as to any conclusion of the exact underlying nature of this aberrant regulation.

In conclusion, our study in control and COPD fibroblasts shows similar regulation of the Smad pathway in COPD and controls without stimulation, but differential effects of particularly cigarette smoke on fibroblast expression of the Smad-genes, an intracellular pathway that is involved in regulation of ECM gene expression. TGFβ, TNF, and CSE cause differential downregulation of decorin gene expression in COPD patients, at least partially via the Smad pathway. Our findings may explain why only a subset of smokers demonstrates an excess parenchymal ECM destruction under influence of cigarette smoking. Smad3, 4 and 7 have to be considered as important factors in the defective repair process of COPD fibroblasts, since smoke exposure affects expression of these genes in COPD but not in control fibroblasts. Because of the chronicity of COPD in combination with its slow progression, even subtle differences in this pathway can have a great impact in the outcome of the disease.

## Abbreviations

ECM: Extracellular matrix; COPD: Chronic Obstructive Pulmonary Disease; TGFβ: Transforming Growth Factor β; TNF: Tumor Necrosis Factor; CSE: Cigarette Smoke Extract; GOLD: Global initiative for Chronic Obstructive Lung Disease; FEV1: Forced Expiratory Volume in 1 second; Ct: Concentration in Time

## Competing interests

The authors declare that they have no competing interests.

## Authors' contributions

AZ carried out the data analysis and drafted the manuscript. DP and WT participated in the design of the original study, were responsible for clinical and histological patient data and contributed substantially to the manuscript. MRJ and AZ carried out real-time PCR. MRJ, JAN and JTWMV carried out the fibroblast isolations and culture and stimulation experiments and contributed to the manuscript.

## Supplementary Material

Additional file 1 Results of real-time PCR analysis 1 and 24 h, GOLD stage II and IV and control. The data provided represent results of real-time PCR analysis at 1 and 24 h, presented for control and disease stage, and per gene of interest and per stimulation. Values are based on 2-delta-delta-Ct values. Click here for file
