# Supplementary material for: Smad gene expression in pulmonary fibroblasts: indications for defective ECM repair in COPD
Source: Respir Res. 2008 Dec 16;9(1):83. doi: 10.1186/1465-9921-9-83 (PMC2613883; doi:10.1186/1465-9921-9-83)
Supplement: Additional file 1 — Results of real-time PCR analysis 1 and 24 h, GOLD stage II and IV and control. The data provided represent results of real-time PCR analysis at 1 and 24 h, presented for control and disease stage, and per gene of interest and per stimulation. Values are based on 2-delta-delta-Ct values. [file 1465-9921-9-83-S1.doc]

**Supplemental Table 1 to:**

**Smad gene expression in pulmonary fibroblasts: indications for defective ECM repair in COPD**

Andre Zandvoort, Dirkje S Postma, Marnix R Jonker, Jacobien A Noordhoek, Johannes TWM Vos and Wim Timens

**Results of real-time PCR analysis 1 and 24h, GOLD stage II and IV and control**

| **1-hour** | |  |  |  |  |  |  |  |
| --- | --- | --- | --- | --- | --- | --- | --- | --- |
|  |  | **Smad 2** | **Smad 3** | **Smad 4** | **Smad 7** | **Decorin** | **Biglycan** | **Versican** |
| **TGF**β | Control | 93 (42-195) | 82 (44-101) | 134 (41-221) | **643 (191-1555)** | 134 (15-169) | 87 (38-158) | 94 (34-137) |
|  | St II | 96 (74-106) | *64 (56-79)* | **119 (97-142)** | **630 (456-831)** | 63 (42-244) | 77 (39-174) | 88 (70-115) |
|  | St IV | 95 (86-110) | *65 (55-74)* | **116 (105-138)** | **585 (384-863)** | 94 (26-147) | *87 (75-102)* | 102 (76-157) |
|  |  |  |  |  |  |  |  |  |
| **TNF** | Control | 101 (91-183) | 95 (89-126) | **106 (103-161)** | 104 (85-104) | 92 (66-146*)* | *69 (55-82)* | 118 (86-156) |
|  | St II | 101 (84-111) | 84 (75-117) | 102 (89-125) | 107 (76-146) | *75 (65-86)* | *50 (39-136)* | 98 (64-158) |
|  | St IV | 106 (86-271) | 82 (70-479) | 101 (86-470) | 108 (83-443) | 83 (79-112) | 65 (53-527) | 99 (60-535) |
|  |  |  |  |  |  |  |  |  |
| **CSE** | Control | 95 (85-104) | 102 (90-112) | 97 (90-100) | 86 (72-120) | *62 (44-86)* | *65 (13-76)* | *69 (25-82)* |
|  | St II | 94 (93-126) | *86 (79-97)* | 98 (87-142) | 107 (83-136) | *65 (50-98)* | 70 (46-153) | 90 (71-115) |
|  | St IV | 103 (93-120) | *81 (69-98)* | 96 (91-115) | 108 (92-145) | 68 (62-382) | *75 (58-95)* | 98 (84-177) |
|  |  |  |  |  |  |  |  |  |
|  | |  |  |  |  |  |  |  |
| **24-hour** | |  |  |  |  |  |  |  |
|  |  | **Smad 2** | **Smad 3** | **Smad 4** | **Smad 7** | **Decorin** | **Biglycan** | **Versican** |
| **TGF**β | Control | **117 (114-131)** | *13 (11-21)* | **116 (103-131)** | **576 (509-1265)** | 62 (42-176) | **228 (186-342)** | **394 (330-784)** |
|  | St II | **111 (99-137)** | *15 (9-22)* | **106 (100-123)** | **742 (608-844)** | *58 (47-70)* | **275 (203-372)** | **474 (284-711)** |
|  | St IV | 106 (89-125) | *15 (13-92)* | 103 (83-126) | **426 (70-996)** | 62 (50-187) | **272 (119-432)** | **570 (164-761)** |
|  |  |  |  |  |  |  |  |  |
| **TNF** | Control | **122 (113-149)** | 104 (99-137) | **111 (105-124)** | **157 (151-272)** | 64 (57-75) | 82 (75-100) | *33 (17-93)* |
|  | St II | **122 (107-177)** | **112 (95-138)** | **113 (99-130)** | **225 (131-344)** | *77 (58-89)* | 98 (86-118) | *68 (46-86)* |
|  | St IV | **116 (100-139)** | 119 (78-141) | 112 (84-155) | 199 (69-281) | 78 (51-219) | 106 (11-122) | 67 (15-202) |
|  |  |  |  |  |  |  |  |  |
| **CSE** | Control | 104 (78-158) | 93 (71-98) | 86 (68-101) | 114 (99-125) | 78 (70-101) | 75 (61-89) | 82 (69-110) |
|  | St II | 99 (66-113) | 110 (93-118) | *93 (82-99)* | **156 (130-189)** | *81 (62-112)* | *86 (48-100)* | 91 (43-123) |
|  | St IV | 89 (79-110) | 107 (11-128) | *89 (81-96)* | **142 (112-381)** | 85 (60-116) | *83 (30-469)* | 87 (47-484) |
|  |  |  |  |  |  |  |  |  |
|  |  |  |  |  |  |  |  |  |
|  |  | **significant upregulation** | |  |  |  |  |  |
|  |  | *significant downregulation* | |  |  |  |  |  |

Results of real-time PCR analysis at 1 and 24h, presented for control and disease stage, and per gene of interest and per stimulation. Values are based on 2^-delta-delta-Ct^ values and represent the median percentages compared to the basal value before each stimulation (basal value set to 100%). Pink and bold indicate significant upregulation of the gene, Purple and italic indicate significant downregulation of the gene. StII = GOLD stage II, StIV = GOLD stage IV.
